# Diversification of Amazonian spiny tree rats in genus *Makalata* (Rodentia, Echimyidae): Cryptic diversity, geographic structure and drivers of speciation

**DOI:** 10.1371/journal.pone.0276475

**Published:** 2022-12-15

**Authors:** Cleuton Lima Miranda, Izeni Pires Farias, Maria Nazareth F. Da Silva, Alexandre Antonelli, Arielli Fabrício Machado, Rafael N. Leite, Mario Da Silva Nunes, Tadeu Gomes De Oliveira, Julio Cesar Pieczarka

**Affiliations:** 1 Postgraduate Program in Zoology of the Museu Paraense Emílio Goeldi, Federal University of Pará, Belém, Pará, Brazil; 2 Laboratory of Animal Evolution and Genetics, Institute of Biological Sciences, Department of Genetics, Federal University of Amazonas, Manaus, Amazonas, Brazil; 3 Mammal Collection, National Amazon Research Institute, Manaus, Amazonas, Brazil; 4 Gothenburg Global Biodiversity Centre, Department of Biological and Environmental Sciences, University of Gothenburg, Göteborg, Sweden; 5 Department of Plant Sciences, University of Oxford, Oxford, United Kingdom; 6 Royal Botanic Gardens, Kew, Richmond, Surrey, United Kingdom; 7 Postgraduate Program in Ecology, National Amazon Research Institute, Manaus, Amazonas, Brazil; 8 Departament of Biology, State University of Maranhão, São Luís, Maranhão, Brazil; 9 Cytogenetics Laboratory, Center for Advanced Biodiversity Studies, Institute of Biological Sciences, Federal University of Pará, Belém, Pará, Brazil; Universite de Pau et des Pays de l’Adour College STEE Sciences et Technologies pour l’Energie et l’Environnement, FRANCE

## Abstract

Amazonian mammal diversity is exceptionally high, yet new taxonomic discoveries continue to be made and many questions remain for understanding its diversification through time and space. Here we investigate the diversification of spiny rats in the genus *Makalata*, whose species are strongly associated with seasonally flooded forests, watercourses and flooded islands. We use a biogeographical approach based on a mitochondrial cytochrome b gene through divergence time estimation and reconstruction of ancestral areas and events. Our findings indicate an ancient origin of *Makalata* for the Guiana Shield and Eastern Amazonia as ancestral area. A first cladogenetic event led to a phylogeographic break into two broader clades of *Makalata* through dispersal, implying a pattern of western/Eastern Amazonian clades coinciding with the Purus Arch (middle Miocene). Most of subclades we infer originated between the late Pliocene to the early Pleistocene, with few recent exceptions in the early Pliocene through dispersal and vicariant events. The hypothesis of rivers as dispersal barriers is not corroborated for *Makalata*, as expected for mammalian species associated with seasonally flooded environments. We identify two key events for the expansion and diversification of *Makalata* species: the presence of geologically stable areas in the Guiana and Brazilian shields and the transition from lacustrine conditions in western Amazonia (Acre system) to a river system, with the establishment of the Amazon River transcontinental system and its tributaries. Our results are congruent with older geological scenarios for the Amazon basin formation (Miocene), but we do not discard the influence of recent dynamics on some speciation events and, mainly, on phylogeographic structuring processes.

## Introduction

### The drivers of Amazonian biodiversity

The profusion of diversification models and taxon-specific results identified for Amazonian organisms reflect the complexity of how Amazonian biodiversity originated and diversified [1]. Amazonia is the largest, oldest, and most biologically diverse rainforest on Earth. For centuries, naturalists have attempted to identify the patterns of this exceptional biodiversity and the processes that shaped the Amazonian biota, postulating multiple diversification scenarios or hypotheses [2, 3]. These hypotheses include the role of rivers as geographical barriers, originally proposed by Wallace and corroborated by several studies involving amphibians, birds and primates [3–5]; and the hypothesis of Pleistocene refuges in driving high and recent speciation [6]. Other hypotheses have been referred to as ecological gradient, disturbance-vicariance, periodic marine incursions caused by eustatic fluctuations in sea level along the Tertiary and structural arches as main geological features causing important barriers in explaining allopatric differentiation [2].

Some of those hypotheses have gained support through the study of some taxonomic groups, but not others; and they have often been found not to be mutually exclusive. For instance, the riverine hypothesis (or more widely, the role of rivers in shaping genetic diversity) has gained support for frogs [7], lizards (e.g., [8, 9]), birds (e.g., [10–12]; but see Santorelli *et al*. [13] for a different result), small mammals [5, 14], primates [15, 16], butterflies [17] and plants [18]. The refugia hypothesis has found some support for plants, butterflies and birds [19–21], although it has been widely rejected based on the mismatch between its recent timing and mechanism and empirical findings (e.g., [22, 23]). The ecological gradient hypothesis has found support from the study of certain trees, lizards and rodents [24]; the disturbance-vicariance hypothesis has been evoked for frogs [25–27]; the marine incursions hypothesis for ants [28] and birds [29]; and the structural arches hypothesis for rodents and marsupials [30, 31] and frogs [32, 33]. This non-exhaustive list clearly shows that Amazonian biodiversity is the outcome of multiple and often intertwined evolutionary processes, where non-deterministic (stochastic) events also can play important roles [34].

### The study group

**Fig 1:**
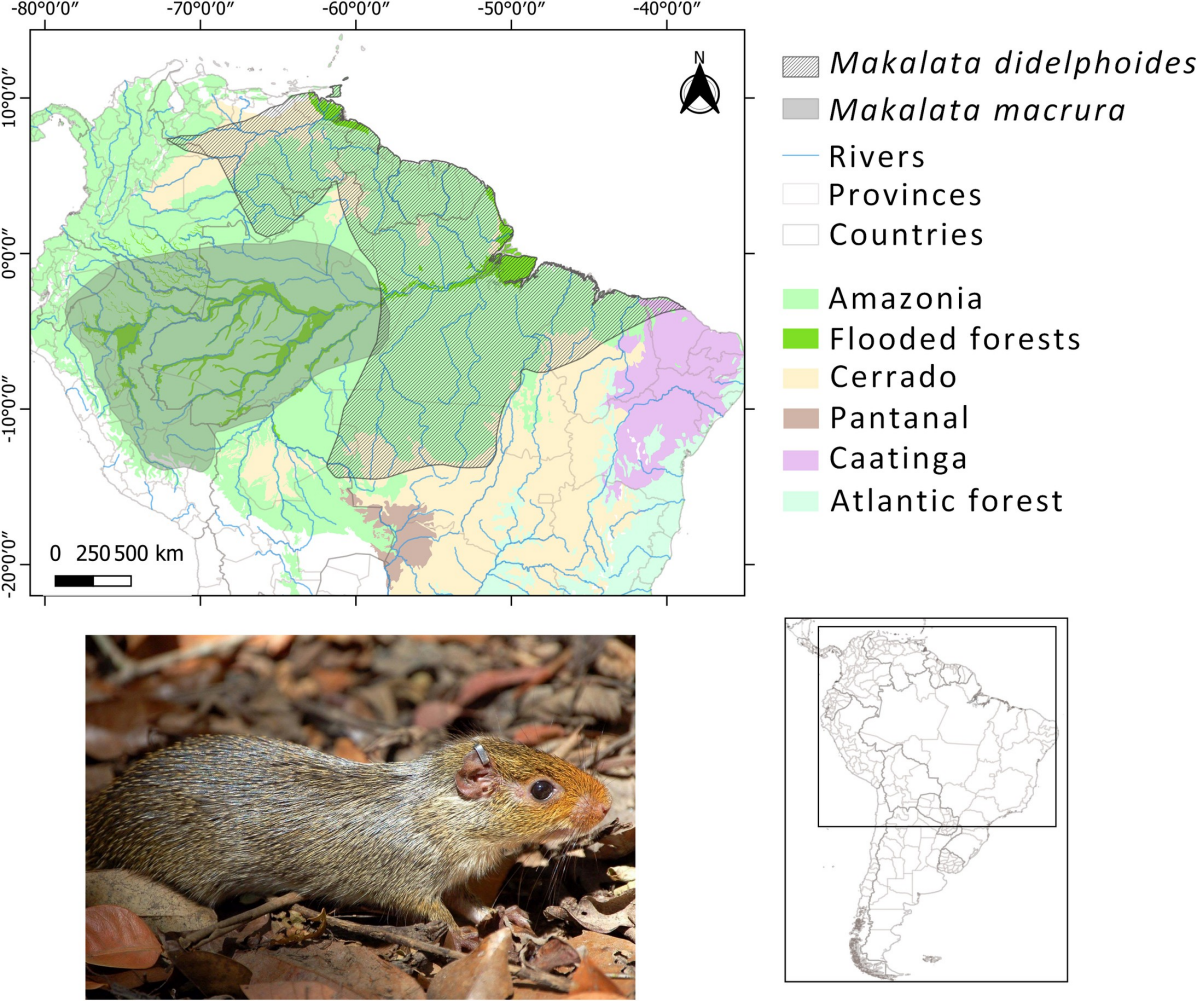
Known distribution for Amazonian spiny tree rat, *Makalata* (Rodentia, Echimyidae). Colours on the map represent the South American environments. Below photo of *Makalata* sp. (head and body length of this species ranging from 237–245 mm for adults). Photo: Rita Rocha.

Our understanding of how biodiversity evolves is dependent on our knowledge of current patterns and how they originated. Since it is likely that the bulk of Amazonian biodiversity comprises inconspicuous and poorly studied organisms such as insects and fungi (e.g., [35]), relatively well-studied groups such as birds, mammals and trees have been the focus of most biodiversity studies [8–10, 12–14]. Even so, a group as charismatic as mammals– arguably the most well-studied of all large groups alongside birds– still contains a significant proportion of poorly studied and even scientifically undescribed species. This challenge is particularly pronounced among rodents, which together make up around 40% of the world’s total mammal diversity, but it still has groups with their biogeography poorly investigated, such as the *Makalata* genus Husson, 1978 – spiny tree rats of the family Echimyidae (Fig 1). *Makalata* species are strongly associated with seasonally flooded forests of Amazonia (várzeas and igapós), where they occur along watercourses and on flooded islands [31, 36, 37].

There are also records of *Makalata* outside the Amazonian domain: in mangrove areas along with three northeast Brazilian states (Maranhão, Piauí and Ceará); in areas of seasonally deciduous forests (“dry forests”) in transition areas between the Cerrado and Caatinga domains; in the Amazonia-Cerrado transition in the north of Tocantins and northeast of Mato Grosso; and in the Amazonia-Pantanal transition of Mato Grosso ([38, 39] Miranda et al., in press; Fig 1). The wide distribution of this group in the Amazonia and adjacent regions, associated with specific environments of seasonally flooded forests or mesic habitats, makes *Makalata* an excellent model system for understanding the evolution of this environment over time.

In addition, *Makalata* has never been extensively revised taxonomically. Its taxonomy remains unstable, with interspecific boundaries poorly known and delineated [31, 36, 39–42]. In the most recent compilation of *Makalata* taxonomy, Emmons *et al*. [41] considered only two valid species for the group: *M*. *didelphoides* (Desmarest, 1817) and *M*. *macrura* (Wagner, 1842). The authors considered *M*. *obscura* (= *Loncheres obscura* Wagner, 1840) as *nomen dubium*. This general lack of knowledge hinders a better understanding of the biogeographic history of the genus and of the processes and mechanisms that led to its diversification.

Several partial taxonomic proposals have been made, comprising different names and numbers of accepted species, ranging from six to nine [43]. The only studies with molecular data for *Makalata* were performed by da Silva & Patton [44] and Patton *et al*. [31], who supported two distinct clades within the genus, referred to as "*didelphoides*" and "*macrura*" by the authors. The “*didelphoides”* clade included three subclades with an average genetic divergence of 11.54%, while the “*macrura”* clade included three subclades with an average divergence of 5.97% [31]. These results highlighted that the taxonomic diversity of the group was clearly underestimated and suggested the need for a broader review of the genus. Recently Miranda et al. (in press) took the broadest approach to delimit potential species for the genus *Makalata* and resolve some outstanding taxonomic issues, such as the applications of the specific names *M*. *didelphoides* and *M*. *macrura*, and re-identifying the karyotypes available in the literature and compiled by Pereira *et al*. [45], in addition to providing two unpublished cytotypes, paving the way for biogeographic studies in the group.

The lack of studies assessing the biogeographic diversification of *Makalata* is in contrast to others available for two closely related genera, *Isothrix* (Wagner, 1845) [46] and *Phyllomys* (Lund, 1839) [42]. However, Upham & Patterson [47] and Upham *et al*. [48] estimated the diversification time in *Makalata*, calibrating the different branches from known fossils and using a relaxed clock technique. The diversification time for the most recent common ancestor of *Makalata* was estimated to approximately 9 million years ago (Mya)–in the middle Miocene. In addition, these authors reconstructed ancestral areas for various genera of echimyid rodents, hypothesizing that the origin of *Makalata* occurred within the Amazonian rather than the Andean region, as found for other groups of echimyids rodents [48].

Given the remaining challenges for our understanding of Amazonian biotic evolution, and Amazonian rodent diversification in particular, the aim of this study is to investigate the biogeography of the genus *Makalata* and the processes and mechanisms that influenced its diversification. To pursue this goal, we infer the temporal and geographical origin of the genus and discuss its diversification in relation to current knowledge about Amazonian biogeography. As this group is strongly associated with seasonally flooded forests and mesic habitats, we hypothesise that rivers have not acted as a strong biogeographic barrier. The biogeography of this group probably has been affected by old events, such as geological, due to the change from the inverse lacustrine system to a transcontinental Amazon River system after the main period of uplift of the Andes [22, 49]. Finally, due to the specific environment used by this group, of flooded forests, our results may also shed light on the biogeographic history of these environments through time.

## Material and methods

### Samples

**Fig 2:**
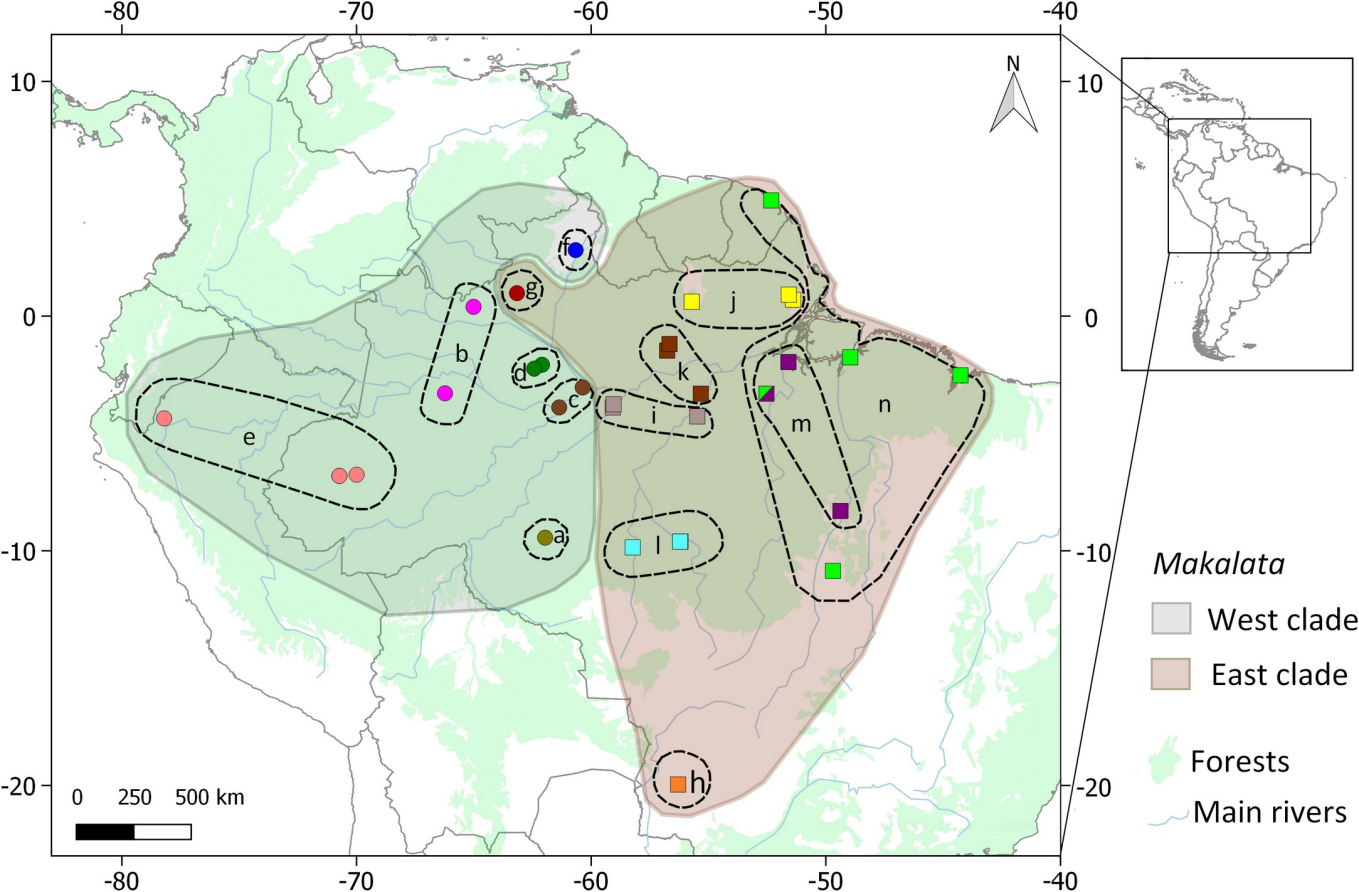
Distribution of the *Makalata* sample collection points sequenced for this study. Localities for two new species of *Makalata* (Miranda et al., in press) are also included.

We used 98 partial sequences (769 bp) of the mitochondrial cytochrome b gene of *Makalata* individuals from 30 locations of *Makalata* (876 bp–amplicon length and 769 after trimming), 68 were produced in the present study, 10 from the Museum of Vertebrate Zoology/INPA sequence database (published in Patton *et al*. [31]) and 26 from NCBI/GenBank (Fig 2 and S1 Table). This research was approved by the Ethics Committee (Animal Research Ethics Committee of Universidade Federal do Pará - Permission 68/2015).

The cytochrome b marker has been widely used in molecular studies of echimyid rodents, including the genus *Makalata* (see [31, 42, 44, 50, 51]), and it has been very informative in the delimitation of lineages and species [52]. Although the use of only one molecular marker has been criticized [53], in particular when inferring deep branches in the tree of life [54], however, several studies have shown sufficient signal to elucidate phylogeographic questions [55], including for small non-flying mammals [5, 56]. The relatively high nucleotide substitution rate is among the many advantages of using mitochondrial markers for biogeographic and phylogeographic analysis [5]. Future studies would profit from the use of high-throughput sequencing techniques to further confirm and refine the results presented here.

### Phylogenetic analysis

Sequences were edited and aligned in Geneious v. 6.1.6 [57], where we also investigated the presence of stop codons. Data saturation was verified through the DAMBE program [58]. Bayesian inference (BI) analyses were performed to assess the phylogenetic relationships among all subclades of *Makalata*. The optimal model of sequence evolution was selected based on the Bayesian information criterion (BIC) using the program jModeltest2 [59] and the information criterion via Akaike: HKY+I +G.

The BI analysis was performed in MrBayes v. 3.1.2 [60]. The analysis consisted of two independent runs of 20^7^ generations, sampling the values every 1,000 generations. We discarded 25% of generations (the ‘burn-in’). We verified convergence of effective sample size (ESS) values considering 200 as a minimum ESS value for all key parameters in Tracer v. 1.6 [61]. We created a consensus tree for BI in TreeAnnotator v. 1.7.5 [61]. Only those groups that presented posterior probability values (pp) equal to or higher than 0.95 were considered significant.

The genetic distance within and between the different monophyletic groupings of *Makalata* that emerged in the consensus tree were calculated using the Kimura 2-parameter model in MEGA v. 6.0 [62, 63]. This model has been the most used in the literature, facilitating comparison between our results and other studies.

We used *Phyllomys blainvilli* (Jourdan, 1837) and *Echimys chrysurus* (Zimmemman, 1780) as external groups. These species were recovered as sister groups of *Makalata* in broader phylogenetic studies that included the Echimyidae family (e.g., [47, 48, 64, 65]). In addition, we used a species of the genus *Proechimys* J. A. Allen, 1899 and *Toromys grandis* (Wagner, 1845) as the most external representative species of the family Echimyidae [47, 48, 65].

We implemented a second Bayesian inference analysis in BEAST v. 1.8.1 [47, 48, 61, 66]. The determination of the evolution model was the same as that used in the phylogenetic analyses. For the construction of the tree we used a pure-birth speciation model (the Yule process), as we did not consider extinction to have played a significant role among these closely related species. We included prior calibrations based on calibration nodes according to the phylogeny of Upham *et al*. [67] with included multiple markers and fossil calibration [48, 67]. The node calibrations were applied for the split between *Makalata* and outgroups (mean = 9.7; stdev = 1.5), for the diversification of sister groups of *Makalata* (mean = 7.3; stdev = 1.5) and for the diversification of *Makalata* (mean = 9.0; stdev = 1.5) considering a normal distribution.

### Reconstruction of ancestral areas and cladogenetic events

We used all post-burn-in trees and the BI consensus tree with the estimation times of each node, obtained in BEAST, to propose hypotheses of ancestral distributions and diversification events. We performed this analysis in RASP v. 4.0 [68] using the BioGeoBEARS package code [69], which tests different models of ancestral range inference, DEC (Dispersal-Extintion-Cladogenesis), DIVALIKE (a likelihood version of Dispersal–Vicariance), and BAYAREAALIKE (a likelihood implementation of the BAYAREA model), and it evaluates the addition of the J parameter to account for founder-event speciation (DEC+J, DIVALIKE+J, BAYAREALIKE+J).

The BAYAREALIKE+J was selected as the best model based on the value of Akaike Weights (AICw) and the AICw from each model can be found in S2 Table. The relative probability of biogeographic areas for each ancestral node is shown in S3 Table and the inference of each diversification event (dispersion, vicariance, and/or extinction) are shown in S4 Table. Our inclusion of a tree sample (rather than single tree) allowed for a comprehensive assessment of the degree of uncertainty of ancestral states [68].

We defined six biogeographic areas frequently used in other studies involving neotropical mammals, which represent well-recognized ecoregions, bioregions, and areas of endemism for Amazonia [70–72]: (A) Eastern Amazonia, situated south of the Amazon River and east of the Xingu River up to the transition areas between Amazonia/Cerrado; (B) Western Amazonia, south of the Amazon River, from the left bank of the Madeira River to Peruvian Amazonia, near the Andean foothills; (C) Guiana Shield, north of the Amazon River and west of the Negro and Branco rivers; (D) Central Amazonia, north and south of the Amazon River, in the northern portion between the Japurá and Negro rivers, and in the southern portion, from the east bank of the Madeira River to the west bank of the Xingu River; (E) Chaco; and (F) Atlantic Forest. We classified the occurrence of each *Makalata* sample in these biogeographic areas and established in which biogeographic area each outgroup occurs, based on distribution maps [73]. Finally, we set a maximum combination limit of four biogeographic areas for any ancestral node to be inferred during the analysis, reflecting the most widespread current species distributions in the genus.

## Results

### Phylogeny

**Fig 3:**
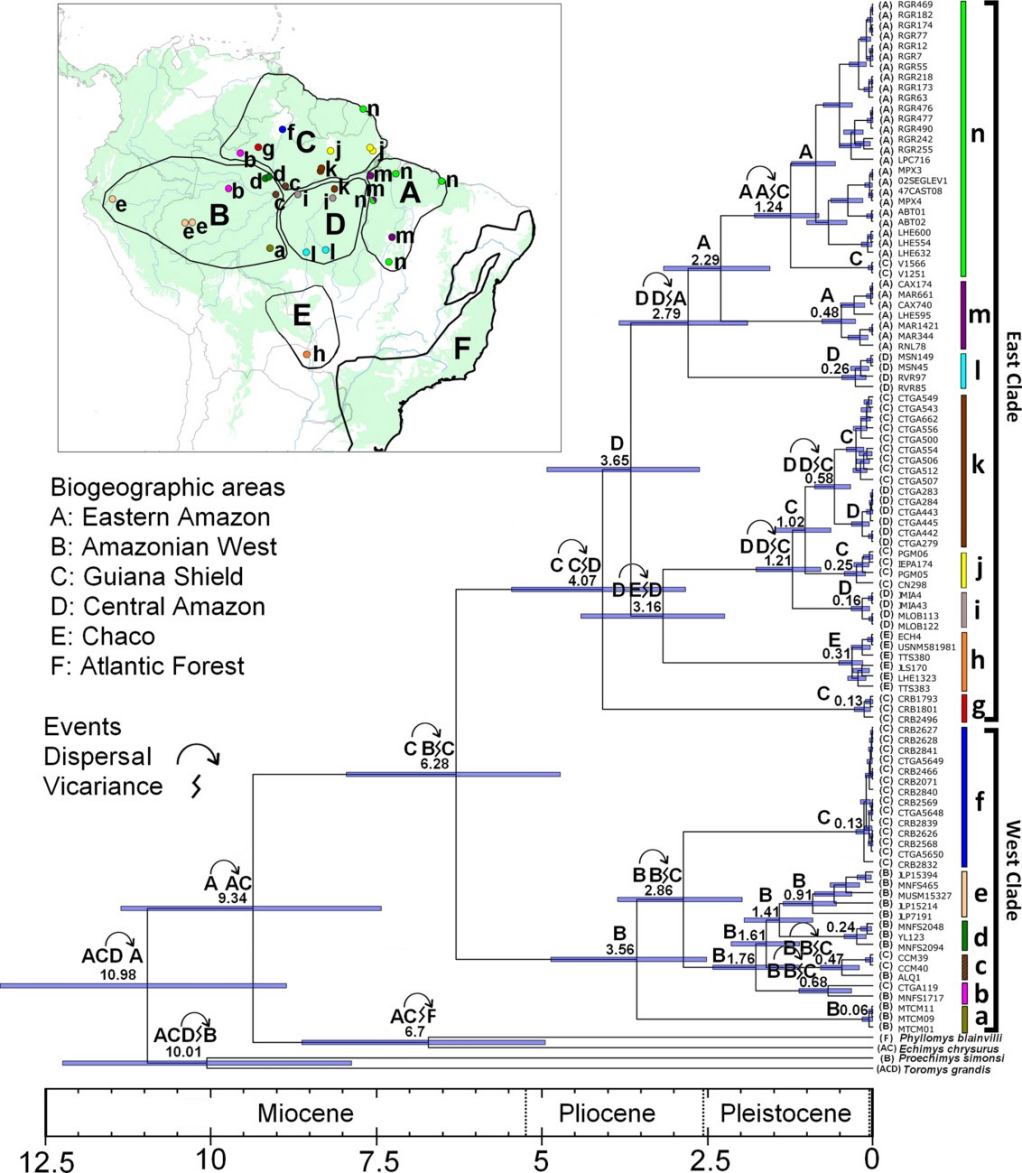
Bayesian divergence time estimation performed in BEAST for *Makalata*, derived from mitochondrial cytochrome b gene. Coloured bars represent subclades recovered in the topology from top to bottom. East Clade: Light green—subclade n = Guiana Shield, Xingu/Tocantins interfluve, Eastern Pará, Middle Araguaia and Maranhão; Purple—subclade m = Xingu/Tocantins interfluve; Light Blue—subclade l = North of Mato Grosso (Juruena and Teles Pires rivers); Brown,—subclade k = Guiana Shield/Trombetas-Tapajós; Yellow,—subclade j = Guiana Shield/Amapá-Northern Pará; Gray—subclade i = Lower Madeira-Upper Tapajos. West Clade (Orange—subclade h = Bolivia; Red—subclade g = Guiana Shield/Negro-Branco rivers; Blue—subclade f = Guiana Shield/Branco River; Beige—subclade e = Juruá River/Peru; Dark green—subclade d = Jaú River; Dashed brown—subclade c = Purus-Anavilhanas; Pink—subclade b = Lower Juruá-Upper Negro rivers; Olive green—subclade a = Upper Jiparaná River. Areas delimited on the map represent biogeographic regions: A = Eastern Amazonia; B = Western Amazonia; C = Guiana Shield; D = Central Amazonia; E = Chaco; F = Atlantic Forest. The arrows represent dispersal events and lightning symbol vicariance.

Our analysis shows that the genus *Makalata* is monophyletic, with high statistical BI support (pp = 1) and a high degree of genetic divergence from external groups (21.8%; Fig 3). The BI topology (Fig 3) consists of 14 distinct evolutionary lineages (subclades) with different karyotypes and all with high values of posterior probability (0.95 to 1). At the base of the topology, two geographically large and highly divergent (18%) clades, named West Clade and East Clade, with high support values (1.0), were recovered.

The West Clade is distributed from the western portion to the Central Amazonia. The East Clade is geographically wider, occurring predominantly in the Eastern Amazonia, including both the north and south of the Amazon River, from the western Guiana Shield (west of the Negro and Branco rivers) to the Amapá coast, stretching south through the left bank of the Tapajós River and its tributaries, the Xingu/Tocantins interfluve and the eastern end of the state of Pará, east of the Tocantins River. Samples belonging to this clade also occur in transition areas between Amazonia and Cerrado in northern Tocantins state (mid-Araguaia River region) and in mangrove areas in northeast Brazil, more precisely in the Maranhão state.

For the West Clade, we retrieved six distinct subclades, with the occurrence of two distinct subclades in the Juruá River region. The average degree of divergence between these subclades ranged from 5.6% to 18.9%, the most divergent being that of the Upper Jiparana River region, Rondônia state. The degree of intra-subclade divergence ranged from 0% to 2% (Table 1).

**Table 1:** Genetic distances between all the 14 subclades recovered in the Bayesian inference topology for *Makalata*. Genetic distances calculated from the Kimura-2-parameter model. Diagonal above represents the standard deviation and diagonal below is the degree of mean divergence. Letters represent each subclade: a = Jiparaná River, b = Juruá-Negro rivers, c = Purus-Anavilhanas, d = Jaú River, e = Juruá River/Peru, f = Guiana Shield/Negro-Branco rivers, g = Guiana Shield/West of the Negro and Branco rivers, h = Bolivia, i = Lower Madeira-Upper Tapajós, j = Guiana Shield/Amapá Northern Pará, k = Guiana Shield/Trombetas-Lower Tapajós, l = North of Mato Grosso (Juruena and Teles Pires rivers), m = Xingu/Tocantins interfluve, n = Guiana Shield-Xingu/Tocantins interfluve-Eastern Pará-Middle Araguaia and Maranhão state.

Subclades/lineages	Genetic distance between subclades														Intra-subclade
	1	2	3	4	5	6	7	8	9	10	11	12	13	14	
**1. N**		1.3	1.3	1.6	1.7	1.5	1.5	1.5	1.8	2	2	1.8	2	2.2	2.5
**2. M**	8.1		1.6	1.6	1.7	1.7	1.7	1.7	1.8	2	2	1.8	2	2	1
**3. L**	8.8	10.4		1.8	1.9	1.7	1.7	1.7	2	2.3	2.2	2	2.2	2.2	0
**4. K**	11.6	10.8	11.6		1	1.1	1.7	1.7	2.2	2	2.4	2.1	2.4	2.5	2.5
**5. J**	11.8	10.7	13.1	5.7		1.1	1.7	1.8	2.4	2.3	2.5	2.2	2.5	0	0
**6. I**	10	11.5	10.8	5.6	5.6		1.6	1.8	2.4	2.3	2.3	2.2	2.5	2.4	0
**7. H**	10	11.4	11.1	11.5	11.3	10.8		1.7	2.1	2.3	2.1	1.9	2.1	2.3	0
**8. G**	10.5	10.9	10.9	11.1	11.2	11.9	11		2	2.2	2.3	2	2.3	2.3	0
**9. F**	13.1	11.9	14.7	15.5	17.9	17.5	15.5	14.3		1.4	1.4	1.3	1.5	1.8	0
**10. E**	16.4	15.4	17.3	16.4	18.1	17.3	16.8	16.4	8.3		1.3	1	1.2	1.8	1
**11. D**	14.7	14.3	16	17.2	13.1	16.6	15.3	16.1	8.3	7.1		0	1.3	2	0
**12. C**	13.8	13.1	15.3	15.9	17.2	16.1	14.1	14.7	7.4	16.1	5.6		0.9	1.7	2
**13. B**	15.7	15.4	16.2	17.8	19.3	18.8	15	16.7	9.3	6.8	7	5.6		1.9	0
**14. A**	18.4	15.1	16.8	18.9	18.3	18.3	17.8	18	11.9	11.9	13.1	11.3	12.8		0

For the East Clade, we recovered eight evolutionary lineages (subclades). The average degree of divergence of these eight clades ranged from 5.6% to 13.1%. The degree of intra-clade divergence ranged from 0% to 2.5% (Fig 3 and Table 1) [41, 43].

### Biogeography and diversification

The Guiana Shield and Eastern Amazonia (AC) were the most likely regions of origin for *Makalata* via dispersal of the common ancestor of this group and its sister group *Echimys* and *Phyllomys* from the Eastern Amazonia to the Guiana Shield (Fig 3, area A to AC, p = 0.58; node 206 in S3 and S4 Tables) around 9.34 Mya (middle Miocene). The probability values (p) refer to the results of the reconstructed ancestral areas with the highest probability, for each node, are available in S3 Table.

Dispersal events followed by vicariance were probably involved in the origin of diversification in *Makalata* resulting in a phylogeographic break of this group into two major clades (West and East clades; at 6.28 Mya) which correspond to the East-West subdivision of Amazonia. This split is most likely to have occurred via dispersal from the Guiana Shield to the Western Amazonia followed by vicariance (Fig 3, area C to BC, p = 0.45; node 204 in S3 and S4 Tables).

From here, we present the cladogenetic events that gave rise to the 14 recovered subclades following the chronological order indicating which of the two broader clades (East and West) this event refers to. As can be seen below, there are older and more recent events interspersed in the two broader clades, East and West, in addition to phylogeographic structuring processes in different subclades, mainly in subclade n in ecotonal regions.

The earlier cladogenetic event in *Makalata* after the phylogeographic break occurred within the East Clade (at 4.07 Mya), giving rise to the group that currently occupies the West Negro and Branco Rivers region (subclade g) via dispersal from the Guiana Shield (C) to the Central Amazonia (D) followed by vicariance (Fig 3, C to CD, p = 0.76; node 203 in S3 and S4 Tables).

Subsequently, around 3.65 Mya, the East Clade diverged into two main clades in Central Amazonia (Fig 3, D, p = 0.50; node 200 in S3 and S4 Tables). The Bolivia subclade (h) was the next to diverge (at 3.16 Mya) through dispersal from the Central Amazonia (D) to the Chaco (E) followed by vicariance (Fig 3, D to ED, p = 0.50, node 162 in S3 and S4 Tables).

The first diversification within the West Clade was dated to approximately 3.56 Mya, resulting in the divergence between the ancestor of the Upper Jiparana River subclade (a) and posteriorly all its subclades in the Western Amazonia (Fig 3, B, p = 0.63; node 134 in S3 and S4 Tables).

The Guiana Shield/Negro-Branco rivers, subclade (f) of the West Clade, emerged around 2.86 Mya, with its ancestor dispersing from the Western Amazonia (A) to the Guiana Shield (C) and becoming isolated by vicariance in the Guiana Shield (Fig 3, B to BC, p = 0.94; node 133 in S3 and S4 Tables).

The subclade (l) named in this study Northern Mato Grosso/Juruena and Teles Pires rivers (East Clade) emerged around 2.79 Mya, most likely due to dispersal from the Central Amazonia (D) to the Eastern Amazonia (A) followed by vicariance in the Central Amazonia (Fig 3, D to AD and D, p = 0.65; node 199 in S3 and S4 Tables). The other subclade of East Clade (Xingu-Tocantins subclade, m) is estimated to have emerged around 2.29 Mya in the Eastern Amazonia (Fig 3, A, p = 0.84; node 195 in S3 and S4 Tables).

The subclade with the largest distribution area, the Guiana Shield/Xingu-Tocantins/East Tocantins River/Araguaia/Maranhão subclade (n) emerged relatively recently (1.24 Mya) through dispersal from the Eastern Amazonia (A) to the Guiana Shield (C) followed by vicariance (Fig 3, A to AC, p = 0.83; node 188 in S3 and S4 Tables).

Subsequently, still in the East Clade (at 1.21 Mya), the Lower Madeira-Upper Tapajós subclade (i) diverged by dispersal from the Central Amazonia (D) to the Guiana Shield (C) followed by vicariance (Fig 3, D to DC, p = 0.54; node 156 in S3 and S4 Tables) resulting in the recent divergence between the Guiana Shield/Amapá-Northern Pará subclade (j) and the Guiana Shield/Trombetas-Lower Tapajós subclade (k) around 1.02 Mya in the Guiana Shield (Fig 3, C, p = 0.73; node 152 in S3 and S4 Tables). Diversification events within the Guiana Shield/Trombetas-Lower Tapajós subclade (k) at 0.58 Mya also showed dispersal and vicariance between the Central Amazonia (D) and the Guiana Shield [C] (Fig 3, D to DC, p = 0.72; node 148 in S3 and S4 Tables).

The Anavilhanas-Purus subclade (c) of the West Clade probably emerged 1.61 Mya by dispersal, most likely in the Western Amazonia [B] (Fig 3, B, p = 0.86, node 117 in S3 and S4 Tables) and diversified through dispersal and vicariance between Western Amazonia (B) and Guiana Shield (C) at 0.47 Mya (Fig 3, B to BC, p = 0.73; node 108 in S3 and S4 Tables).

Still in the West Clade, the Juruá subclade (b), which includes samples of the Guiana Shield (C) and Western Amazonia (A), emerged approximately 1.76 Mya in Western Amazonia [A] (Fig 3, B, p = 0.94; node 115 in S3 and S4 Tables) and diversified through dispersal and vicariance between Western Amazonia (B) and Guiana Shield (C) at 0.68 Mya (Fig 3, B to BC, p = 0.73; node 108 in S3 and S4 Tables).

The Jaú River subclade (d) of the West Clade emerged around 1.41 Mya in Western Amazonia [B] (Fig 3, B, p = 1; node 110 in S3 and S4 Tables) and, finally, the Juruá/Peru subclade (e) emerged around 0.91 Mya in Western Amazonia (Fig 3, B, p = 1; node 114 in S3 and S4 Tables).

## Discussion

### Phylogenetic relationships

Our results corroborate the presence of two well-differentiated clades within *Makalata*–the West and East clades which were already revealed by Patton *et al*. [31]–while increasing the number of recognized lineages from six to 14. The West clade partially corresponds to the “*macrura*” clade described in Patton *et al*. [31], since four of the six subclades that emerged in our topology had already been presented by these authors, but with the inclusion of new sequences in the present work. The East Clade corresponds to the “*didelphoides*” clade described in Patton *et al*. [31], for which we present six new subclades in addition to the two previously presented in that study.

### Divergence time [31, 45]

The estimated time for the most recent common ancestor of the genus *Makalata* and its sister group (*Phyllomys* and *Echimys*) is 9.3 Mya, which represents the temporal origin of the genus *Makalata* (i.e., its crown group). This result is based on the mitochondrial cytochrome b gene and is similar to that found by Upham *et al*. [48] at 9 Mya, estimated using five molecular markers: two mitochondrial, one of which was cytochrome b and three nuclear genes. However, it is important to note that this study included one or two taxa representing each echimyids genus. For *Makalata*, two samples were identified as *M*. *didelphoides* and *M*. *macrura* in that study, representing an interspecific approach [31]. Therefore, the present study represents the approach with the densest sampling in relation to the inclusion of samples and geographic coverage for *Makalata*, bringing a novel intraspecific approach to assessing its biogeographic history. Nevertheless, both these estimates agree that *Makalata* origin correspond to the middle Miocene.

According to Hoorn *et al*. [22], the middle Miocene was a very dynamic time, with drastic climate and topographic changes triggered by a strong uplift phase of the Andes mountain range, especially in its northern portion [74]. This period also corresponds to a shift from a lacustrine system (Acre system) to a transcontinental Amazon drainage, implying changes in the flow direction and size of the current Amazonian river system, as well as expansion of the area covered by seasonally flooded forests (várzeas and igapós) and Terra Firme Forests [75–78].

Also according to different authors [22, 79–81] at around 8 Mya the Amazon River consisted of two sub-basins, Solimões (west) and Amazonas (east) bounded by a geological structure known as Purus Arch, located west of Manaus, in Central Amazonia. This Arch probably originated and served as a divider between these two independent basins [82], providing numerous floodplains areas in the western and Eastern Amazonia that must have been excellent facilitators for the dispersal events in *Makalata*. The subsidence of the Purus Arch transformed the lacustrine system of the great Amazon Lake into a riverine Amazon River system. The formation of the Amazon River and some of its tributaries completed between 3 and 7 Mya [22]. Consequently, this time appears to have been important for the generation of biological diversity in the Amazonia [3, 77].

However, there is no consensus on the events and scenarios regarding the formation of the transcontinental Amazon fluvial system, especially the timing when such events would have occurred. For instance, Latrubesse *et al*. [83] suggested the beginning of the Pliocene (about 5 Mya) as the time when such a system would have been completed, presenting a conformation like the current one. In general, two lines of thought regarding geological models for the Amazon can be considered: the first that proposes an older dynamic for the formation of the Amazon basin, which includes from the middle Miocene to the beginning of the Pliocene [22, 83], and another which suggests a more recent dynamic with several events between the Pliocene/Pleistocene (e.g., [10, 84, 85]). The most recently dated model was proposed by Aleixo *et al*. [85], which points out that the formation dynamics of the transcontinental system would have been completed only in the Pleistocene. However, Hoorn *et al*. [79] identified various misinterpretations of such scenario, highlighting the lack of undisputable evidence and scientific consensus in this subject. We consider that there is a greater amount of evidence to support the hypotheses of Hoorn *et al*. [79].

### Biogeography

**Fig 4:**
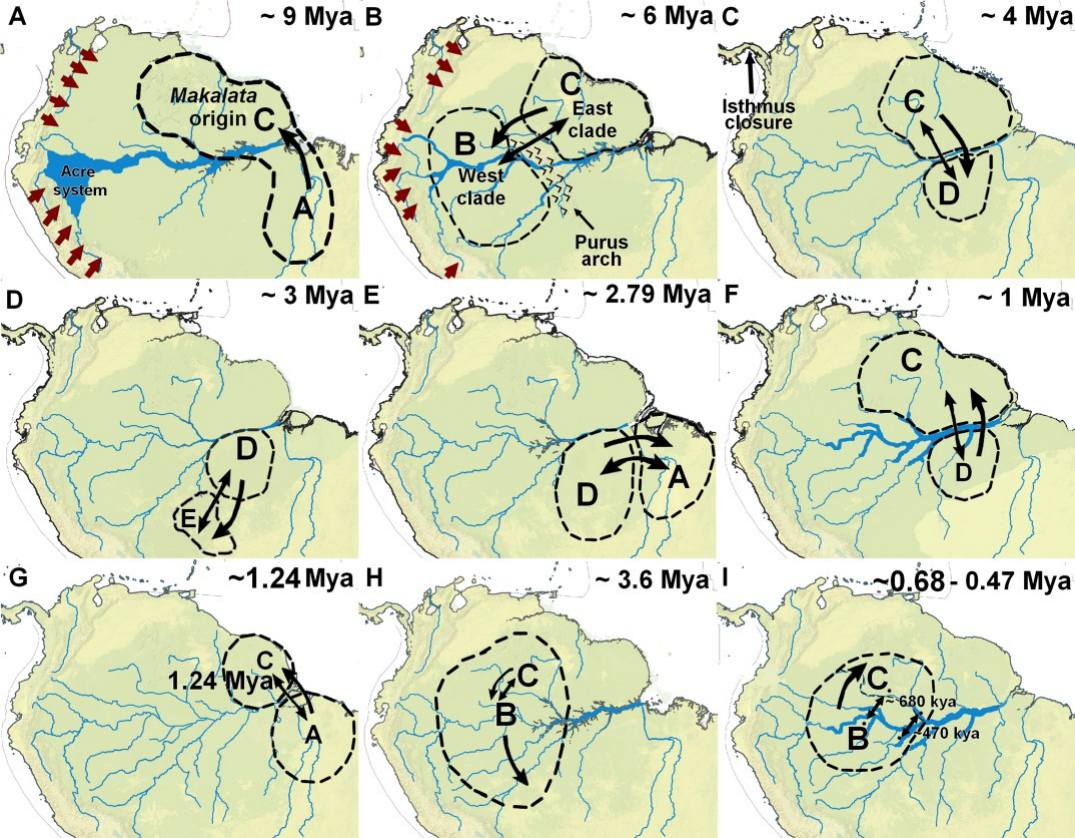
Main *Makalata* biogeographic diversification events. The numbers indicate the sequences of events in chronological order and the capital letters in parenthesis the biogeographic regions delimited for the analysis. (A) Origin of *Makalata* ancestor via dispersal (black arrow) from the Eastern Amazonia to the Guiana Shield during the middle Miocene after Andean Uplift (brown arrows) and transition of the fluvial system (Acre system) to the transcontinental drainage system; (B) First break within *Makalata* (West and East clades) by dispersal between the Purus Arch followed by vicariance; (C) First and second splits in the East Clade by dispersal and vicariance between the Guiana Shield and the Central Amazonia during the early Pliocene; (D) Dispersal of the Bolivian subclade (h) ancestor from the Central Amazonia to the Chaco geographic region; (E) Vicariance from the Chaco region to Central Amazonia in the late Pliocene and dispersal of the ancestor of the subclades l, m and n from the Central Amazonia to the Eastern Amazonia; (F) Dispersal of subclades i, j and k ancestor from the Central Amazonia to the Guiana Shield during the early Pleistocene marked by the trunked Amazon River; (G) Diversification of the subclade n through dispersal from the Eastern Amazonia to the Guiana Shield followed by vicariance during the Early Pleistocene; (H) First split within the West clade by dispersal of the Jiparana subclade ancestor from the western portion of the Guiana Shield to the Western Amazonia followed by vicariance; (I) Diversification of the subclades b and c in the early Pleistocene through dispersal from the Western portion of the Guiana Shield following by vicariances events between Western and Central Amazonia during the late Pliocene (subclade b in ~680 kya and subclade c ~470 kya).

Our results point to the Guiana Shield and the Eastern Amazonia as the potential region of origin and diversification of the ancestor of *Makalata* and its sister group (*Phyllomys* and *Echimys*) via dispersal (Fig 4A and Table 2), and not the Andean region, like for other current genera of echimyid rodents [48]. In fact, this whole region represented by the Northern Amazon hosts some very ancient lineages associated with tepuis (see e.g., [86]). Another previous study sought to reconstruct ancestral areas of neotropical caviomorph rodents (superfamily Octodontoidea) including some echimyid rodents [45]. Although that study estimated the ancestral area of *M*. *didelphoides* (East Clade) to the Atlantic Forest, this is now considered an error, related to the incorrect estimation of the phylogenetic relationships of *Makalata* and *Phyllomys*. This was later resolved in Upham *et al*. [67]), where Amazonia region was inferred as ancestral area for *Makalata*. Here we corroborate the results of that later study [65], however, our broader approach reveals that the group arose through dispersal between these specific regions of northern and Eastern Amazonia.

**Table 2:** Diversification events of *Makalata* clades (East and West) and subclades (a -n) found in our study.

Diversifying groups	Node age	Heigth 95% HPD	Geological period	Event	Ancestral area	Key events
*Makalata* origin	9.34	7.41–11.38	Mid-Miocene	dispersal	ABCD->BCD	Acre system. Geologically stable areas in the Guiana Shield and the Brazilian Shield.
West Clade* *versus* East Clade*	6.28	4.71–7.94	Late Miocene	dispersal	BCD	Purus Arch. Transition from the Acre system to the Amazonas/Solimões river system.
subclade g *versus* subclades h, i, j, k, l, m and n	4.07	2.82–5.45	Early Pliocene	dispersal	CD	Decreasing sea level, GABI and quaternary oscillations.
subclades h, i, j, k *versus* subclades l, m, n	3.65	2.61–4.91	Early Pliocene	dispersal	CD	Rising sea level
subclade a *versus* subclades b, c, d, e and f	3.56	2.51–4.85	Late Pliocene	dispersal	BC	Decreasing sea level
subclade h *versus* subclades i, j and k	3.16	2.23–4.40	Late Pliocene	dispersal and vicariance	CD	Isolation by open vegetation (Chaco, Cerrado and ecotones)
subclade f *versus* subclades b, c, d and e	2.86	1.97–3.85	Late Pliocene	dispersal	BC	Diversification and expansion. Emergence of favorable flooded forest habitats and Quaternary oscillations.
subclade l *versus* subclades m and n	2.79	1.89–3.83	Late Pliocene	dispersal and vicariance	AC	Diversification and expansion. Recent dynamics of the mouth of the Amazon River and Quaternary oscillations.
subclade m *versus* subclade n	2.29	1.55–3.16	Early Pleistocene	dispersal	AC	Quaternary oscillations. Relictual mesic environments in east distribution. Diversification and expansion.
subclade b *versus* subclades c, d and e	1.76	1.20–2.41	Early Pleistocene	dispersal	BC	Emergence of favorable flooded forest habitats. Diversification and expansion.
subclade c *versus* subclades d and e	1.61	1.09–2.14	Early Pleistocene	dispersal	B	Emergence of favorable flooded forest habitats. Diversification and expansion.
subclade d—subclade e	1.41	0.91–1.94	Early Pleistocene	dispersal	B	Emergence of favorable flooded forest habitats. Diversification and expansion.
subclade i *versus* suclades j and k	1.21	0.79–1.76	Early Pleistocene	dispersal	CD	Recent dynamics of river orogeny and favorable habitats in the Guiana Shield. Quaternary oscillations. Diversification and expansion.
subclade j *versus* subclade k	1.02	0.64–1.48	Early Pleistocene	dispersal	CD	Recent dynamics of river orogeny and favorable habitats in the Guiana Shield. Quaternary oscillations. Diversification and expansion.

*West Clade: a = Jiparana River, b = Juruá-Negro rivers, c = Purus-Anavilhanas, d = Jaú River, e = Juruá River/Peru, f = Guiana Shield/Negro-Branco rivers. *East Clade—M. didelphoides: g = Guiana Shield/West of the Negro and Branco rivers, h = Bolivia, i = Lower Madeira-Upper Tapajos, j = Guiana Shield/Amapá Northern Pará, k = Guiana Shield/Trombetas-Lower Tapajós, l = North of Mato Grosso (Juruena and Teles Pires rivers), m = Xingu/Tocantins interfluve, n = Guiana Shield, Xingu/Tocantins interfluve, Eastern Pará, Middle Araguaia and Maranhão.

The first phylogenetic split within the ancestor of *Makalata* and sister groups (*Echimys* and *Phyllomys*) would have occurred in the Eastern Amazonian portion, a geologically more stable region and probably with a river conformation closer to the current one, with availability of habitats favourable to *Makalata* diversification, either by dispersal, vicariance, or both. At this time, most of the lowland area of the Western Amazonia would still have been occupied by the Acre system when this group diversified ([22, 86, 87]). Some studies [83, 88] suggested a larger extension to the Acre system at this time, reaching approximately 300 km from Manaus, Amazonas, near the Purus Arch. Therefore, it seems more parsimonious to think of an ancestral area for *Makalata* in the Eastern Amazonia, with the presence of seasonally flooded forests in a transcontinental drainage system established between 12 and 7 Mya, or even ca. 5 Mya, as suggested by Latrubesse *et al*. [83] (but see [22, 79]).

The Guiana and Brazilian Shields are considered geologically older and more stable regions, when compared, for example, with Western Amazonia [22]. In this respect, our results are not congruent with the most recent timing proposed for the establishment of the Amazon basin (e.g. [10, 84]), being more in accordance with older estimation [22, 79, 80, 83, 87].

From the Guiana Shield, many dispersal events and some vicariant events led to the diversification of *Makalata* and to the establishment of populations both west and east of Amazonia, only recently reaching transition areas between Amazonia/Cerrado and mangroves in Northeast Brazil, such as discussed below.

The first major phylogeographic break for *Makalata* was estimated at approximately 6.28 Mya at the Late Miocene by dispersal followed by vicariance, implying an east–west Amazonian distribution pattern (Fig 4B and Table 2). A similar biogeographic pattern has been found for other wildlife groups, such as lizards [77, 89–93], amphibians [94]; birds [95, 96] and mammals [44, 97].

The Guiana Shield were the most likely region for this first phylogeographic break within *Makalata* (Fig 4B and Table 2). This region was close to the location of the Purus Arch [4], and this geological arch has already been suggested as a boundary between the western and Eastern Amazonia [49, 80].

Geological arches have been a widely discussed topic in the literature, with different points of view (e.g. [4, 30, 49, 78, 98]). Studies reported the coincidental position of phylogeographic breaks of extant mammals (and anurans) along the Jurua River in western Brazil with that of the structural Iquitos Arch and of the timing of sub-basin formation on either side of the arch (between middle Miocene to Pliocene), and clade formation estimated from molecular divergences [78]. Yet, Rossetti *et al*. [98] argue that the association of these arches as geographical barriers to modern species were not sustained, as they were buried by very old sediments dating from the Cretaceous and Cenozoic [49]. However, Figueiredo *et al*. [88] proposed a more recent reactivation for the Purus Arch during the Miocene, a time coincident with the phylogeographic break we observed for *Makalata* and already observed for several other faunistic groups.

Thus, the Purus Arch may have played a role in determining the biogeographic pattern we observed for *Makalata* (phylogeographic split for 6.28 Mya by a dispersal event). Between 7 and 5 Mya this geological structure supposedly no longer divided the Amazon drainage into two sub-basins [22, 83], implying the transition from a lake system to a river system, with the gradual decrease of the area occupied by a lake system in the western Amazonia and emergence of flood habitats favourable to the group’s expansion. This transition may have played a key factor in *Makalata*’s expansion and diversification (Fig 4B and Table 2). Studies indicate that these arches would be structures much older than *Makalata* and even though some authors point to the reactivation of the Purus arch, its role for biological diversification in that region is not clear. Our own results indicate that the break was due to dispersal and not vicariance. However, the possibility of influence of this geological structure on important factors such as vegetation or river formation cannot be ruled out.

Among most subclades of *Makalata*–which represent different putative species according to Miranda *et al*. (*in press*)– the oldest one (first diversification within the East Clade) emerged at the beginning of the early Pliocene (4.07 Mya) in the Guiana Shield through dispersal to the Central Amazonia followed by vicariance, representing the diversification of the subclade g from West of the Negro and Branco rivers (Fig 4C and Table 2). The second oldest divergence (first diversification within the West Clade) occurred during the late Pliocene (3.56 Mya) through dispersal from the western Guiana Shield to the Western Amazonia followed by vicariance, representing the diversification of the subclade a from Jiparana River (Fig 4H and Table 2). Vicariant events after dispersal were involved in both cladogenetic events, suggesting ancient expansion from the Guiana Shield to the southern Amazonia, probably occupying flooded forest areas specially for the Jiparana subclade.

The distribution of older subclades clearly shows the diversification of the group into more stable areas and supposedly with available habitats. The Upper Jiparana and Bolivia subclades (probably bordering the areas close to the Acre system in an intense process of retraction), as well as the subclades that emerged at the end of the Pliocene in the Guiana Shield, clearly point to this hypothesis. Another important point would be the emergence of very recent lineages and even more recent returns of lineages from the western and Central Amazonia to the north of the Amazon River, in the Guiana Shield. Furthermore, the strong phylogeographic structure of subclade n, which has the widest distribution, arrived recently, and established itself in mesic environments in the Amazon-Cerrado-Caatinga ecotonal region.

Another important biogeographic event, known as Great American Biotic Interchange (GABI), may also have influenced the dispersal events of the *Makalata* subclades after the closing of the Isthmus of Panama. This event led to the migration of many taxa between North America and South America through the Isthmus of Panama [99, 100]. Among these taxa were, for example, sigmodontinae rodents, which arrived from the north and expanded rapidly across the South American continent after their invasion [101]. Thus, the arrival of these new taxa occupying the environments previously restricted to South American taxa, and then competing for these new niches, may have also driven the dispersal of *Makalata*’s ancestral populations to the Southern Amazon during this period.

[22, 86] Still in the Pliocene, around 3 Mya, another diversification also showed expansion independently from the Central Amazonia but here together with the Guiana Shield (Fig 4D and Table 2), more specifically to the region that currently corresponds to the Bolivian Chaco. This region is covered typically by open environments but also riparian forests, and it was colonised through dispersal followed by vicariance. The transition from forest to open environments of this wide region (represented by the Moist Chaco and Pantanal) may have been the vicariant event responsible for the origin of the Bolivia subclade.

Most diversification events that led to speciation in *Makalata* occurred in the late Pliocene/early Pleistocene, considering confidence intervals. Only two lineages diversified at the beginning of the Pliocene, both were earlier branching within the broader clades (West and East) recovered in the present study and discussed earlier (Guiana Shield/East Negro and Branco rivers and Upper Jiparaná River subclades). According to Hoorn *et al*. and Latrubesse *et al*. [22, 83], by the end of the Pliocene/early Pleistocene the landscape and conformation of the Amazonia region was already more similar to the present one, with all the major rivers supposedly already established, although some with somewhat different conformation from the present day ([22, 80]). This scenario differs from that proposed by other authors, who propose much more recent timings (between 3 Mya until very recently) [10, 98, 102].

These subclades would have diversified independently within the West and East clades, with Western-clade-related lineages expanding and diversifying towards the Western Amazonia, and the East clade lineages or subclades further south-east, reaching the transition areas between Amazon/Cerrado and Northeast Brazil (as for the divergence of the Eastern Amazonia during 2.79 Mya, Fig 4E and Table 2). Cladogenetic events appear related to both dispersal and vicariance. There are also potential sympatric regions for different subclades (West and East) near the Manaus region and the Upper Negro River region. Furthermore, the samples for the Purus-Madeira interfluve are scarce and it is not possible to rule out this possibility also for that region. These areas of sympatry would have recently been established involving dispersal from the Western Amazonia to the Guiana Shield, followed by isolation by vicariance, which would not have led to speciation but intraspecific geographical structuring (subclade b Juruá and subclade c Purus-Anavilhanas, Fig 4I and Table 2).

### Comparison with other groups

Clade diversification in *Makalata* (Miranda *et al*., in press) was more recent than that inferred for species of *Phyllomys*, an Atlantic Forest echimyid genus closely related to *Makalata* (e.g. [47, 48, 66]). While for *Makalata*, most species appeared between the end of the Pliocene and the beginning of the Pleistocene (considering the broader confidence intervals we obtained in our study), for *Phyllomys* most of them appeared earlier, at the end of the Miocene/early Pliocene (5.56 Mya). However, as for *Makalata*, some *Phyllomys* species are also inferred to have diversified more recently in the late Pliocene and early Pleistocene [42, 51].

It is interesting to compare the origins and diversification times found for *Makalata* and the squirrel monkeys of the genus *Saimiri* Voigt, 1831 [16], as both genera are adapted to seasonally flooded environments. While *Makalata* results are congruent with an older dating for the formation of the Amazonian drainage, Saimiri’s are consistent with more recent origins. As we described, *Makalata* probably originated in the Guiana Shield/Eastern Amazonia in the Miocene (9 Mya), while the origins of *Saimiri* are inferred for the Southeast Amazonia, in the region where the Rondônia and Inambari centres of endemism (1.6 Mya) are today. We postulate that *Makalata* diversified independently within two major clades, both in western and Eastern Amazonia (between 9 and 1.24 Mya), but *Saimiri* diversified first towards the western and the Central Amazonia and later to the Eastern Amazonia. Similar to the scenario proposed by Lynch Alfaro [16] for *Saimiri*, we also attribute the transition from a lacustrine to a river system, with subsequent expansion and diversification events by riparian corridors, as key events for the diversification of *Makalata*. However, the estimated times and directions of expansion and diversification of the faunal groups in question are distinct.

Considering their strong association with flooded forests (várzeas and igapós) in the Amazon, *Makalata* species cross large rivers such as the Amazon, Madeira, Tapajós, Xingu and Tocantins rivers–potential geographical barriers for other non-faunal groups. Thus, for most clades, *Makalata* diversification patterns do not support the hypothesis of rivers as barriers [30, 78, 103, 104]. According to Aleixo [105], species that specialize in these types of environments have greater capacity to disperse across islands or even cross rivers [30], an observation corroborated by our results for *Makalata*. Consequently, the distribution of clades recovered in our topologies suggest that the current proposed endemism centre, which are based mainly on results for various bird groups (e.g. [10, 106, 107]), may not reflect patterns in other groups.

## Conclusions

In the present study, we identified 14 subclades in the genus *Makalata* using molecular and cytogenetic data. Overall, our results are congruent with the influence of older and intermediate geological scenarios on the diversification of the species in the genus, since most cladogenetic events are estimated to have occurred before the end of the Pliocene in more geologically stable regions, rivers with more similar conformation to the current one, and supposedly favourable habitats for diversification (e.g., várzeas and igapós). However, estimates of diversification for some subclades were placed between the end of the Pliocene and beginning of the Pleistocene. These two findings reinforce the idea that Amazonian diversification as a whole has old origins and continuous diversification [22, 108]. Moreover, we found strong phylogeographic structuring during the Pleistocene, which could be related to the most recent changes in the shape of the rivers and landscape that make up the Amazon basin. Alternatively, genetic differentiation during this period may have been the result of stochastic dispersals across barriers, after the river systems had already been fully formed [34]. In both cases, these results indicate that the Pleistocene was an especially important period for phylogeographic expansion and structuring of *Makalata*, with its arrival in both the far east and west of its current distribution. This pattern of more recent lineages in the western and eastern limits of the genus’ distribution appears linked to the dynamics of retraction of the Acre system (west) and effects of the oscillations of the Quaternary (east).

## Supporting information

S1 Table Localities gathered in the present study for the genus *Makalata* (tissues and/or karyotypes). CTGA = vouchers collected by some authors of this work that will be deposited in the INPA mammal collection, Amazonas; MLOB 122 (INPA 6781) and MLOB 133 (INPA 6800); CRB = Cibele Rodrigues Bonvicino and will be deposited in the collection of mammals of the MN, Rio de Janeiro; MTCM = vouchers will be deposited in the mammal collection of the MZUSP, São Paulo; RVR = vouchers will be deposited in the mammal collection of UFMT; the other acronyms are used in the work of Patton et al. (2000) in the table 53, pg. 190. Some sequences (10) in the paper by Patton et al. (2000) could not be deposited with Genbank and are marked with an asterisk and listed as not deposited. Menezes et al. (2007): The sequences of this article were deposited with Genbank but the article was not accepted and not posteriorly published in another journal (C. R. Bonvicino, personal communication).
(XLSX)

S2 Table Results of the model test for reconstruction of *Makalata* ancestral area. (XLSX)

S3 Table Complete probability values of ancestral reconstruction areas for each node of phylogenetic Bayesian inference of *Makalata* using BAYAREALIKE model in RASP. (XLSX)

S4 Table Probability values of dispersal and vicariant events for each node of phylogenetic Bayesian inference of *Makalata* using BAYAREALIKE model in RASP. (XLSX)
